# Nonlinear dynamic stability of piezoelectric thermoelastic electromechanical resonators

**DOI:** 10.1038/s41598-020-59836-0

**Published:** 2020-02-19

**Authors:** Masoud SoltanRezaee, Mahdi Bodaghi

**Affiliations:** 1 Department of Mechanical Engineering, Tarbiat Modares University, Tehran, Iran; 2 Department of Engineering, School of Science and Technology, Nottingham Trent University, Nottingham, United Kingdom

**Keywords:** Mechanical engineering, NEMS, Nonlinear phenomena

## Abstract

This research work deals with analyzing instability and nonlinear behaviors of piezoelectric thermal nano-bridges. An adjustable thermo-elastic model with the ability to control stability conditions is developed to examine the system behavior at different temperatures. To increase the performance range and improve system characteristics, a piezovoltage is applied and a spring is connected to the sliding end of the deformable beam as design parameters. The partial differential equations (PDEs) are derived using the extended Hamilton’s principle and Galerkin decomposition is implemented to discretize the nonlinear equations, which are solved via a computational method called the step-by-step linearization method (SSLM). To improve the accuracy of the solution, the number of mode shapes and the size of voltage increments are analyzed and sufficient values are employed in the solution. The validity of the formulation and solution method is verified with experimental, analytical, and numerical data for several cases. Finally, the vibration and eigenvalue problem of the actuated nano-manipulator subjected to electrostatic and Casimir attractions are investigated. It is concluded that the fringing-fields correction changes the system frequency, static equilibrium, and pull-in characteristics significantly. The results are expected to be instrumental in the analysis, design, and operation of numerous adjustable advanced nano-systems.

## Introduction

Recently, micro/nano-electromechanical systems (M/NEMS) have attracted numerous attentions due to their ultra-small dimensions, favorable mechanical and electrical properties, and low power consumptions. These tiny structures represent the most promising contenders in numerous micro/nano-based applications in several fields. As a result, modelling and analysis of pull-in characteristics in the micro-scale have found significant industrial applications. However, as the pull-in instability restricts the operational range of tiny devices, obtaining system characteristics becomes important in numerous micro/nano-structures^1–11^.

In general, classical theories are not able to model the real behaviors of electromechanical systems in the nano-scale. That is because several effects, especially intermolecular interactions play significant roles in the submicron-scale. To predict the accurate response of different NEMS structures, several theoretical models have been developed and modified. For example, electric fringing-fields corrections (FFC)^12,13^, dispersion forces (Casimir and vdW)^14,15^, size-dependent theories (couple stress (CST), strain gradient (SGT), and so on)^16,17^, and surface layer elasticity models^18,19^ have been taken into account so far. Each of these models and theories has special applications based on the miniature system properties and circumstances. It has been demonstrated that without taking the fringing corrections and dispersion effects into account, the structure will behave harder^14,20,21^. As a result, the predicted NEMS responses may not be accurate enough. Moreover, the impacts of size-dependency and surface layer energy can be a hardening or softening factor at different conditions in submicron structures^21–23^. The role of all these effects on the behavior and stability of nano-systems will be considered and discussed by a set of parametric studies in this paper.

In order to be able to overcome the problems of conducting conscientious experimental examinations, researchers have been competing to improve the modelling tools capable of predicting the behavior of different types of NEMS. One of the most important issues is the electrostatic force, which acts an essential role in the pull-in instability of micro/nano-systems. On the other hand, it has been demonstrated that the electric FFC is an effective nonlinearity in investigating actuated small-scale systems^24,25^. Recently, the impacts of FFC on the response of several fully-clamped nano-beams have extensively been examined^20^. Ouakad^20^ found that FFC should be taken into account because this consideration improves the prediction of the critical voltage for the snap-through, and the overall behavior of arch nano-systems. Moreover, the experimental validation demonstrates that the fringing correction in nano-devices acts a key role that should be considered in the modelling.

Thermal sensitive nano-electromechanical manipulators have numerous industrial applications. Rokni and Lu^26^ studied the behavior of fully-clamped multi-layer nano-ribbons subjected to electric and Casimir forces at different temperatures without considering the size parameter. The stability of fully-clamped miniature beams under thermal, molecular and electromechanical effects was examined^27^ without modelling the surface energy. The static pull-in instability of a thermal nano-cantilever piezowire under vdW attraction was examined by SoltanRezaee and Ghazavi^28^ while the frequency analysis and investigation of different electric fringing-fields corrections were not carried out. Pradiptya and Ouakad^29^ investigated the size-dependent dynamic response of fixed-fixed carbon nano-tubes subjected to DC and AC fields by considering thermal effects using SGT. The impacts of Poisson’s ratio on structural behaviors of a nano-wire by considering high-temperature variations were studied using CST^30^. Recently, Tavakolian *et al*.^31^ analyzed the dynamic stability of size-dependent nano-cantilevers under electro-thermal actuation without taking the surface layer effects into account. It is worth noting that the pull-in parameters of clamped-clamped smart MEMS were extensively studied^32–34^; however, with numerous practical applications of micro-beams with the sliding end^35,36^, they have been less attentive. Overall, it can be concluded that temperature variation affects the instability considerably that should be taken into account.

In this research, the coupled contributions of molecular forces, surface layer energy, size-dependency, piezoelectricity, FFC, thermal expansion, and geometrical nonlinearity on the pull-in characteristics using modified CST and SGT are considered for the first time. All the mentioned parameters represent key roles in piezoNEMS and it is remarkable to account their effects on both static and dynamic results of nano-bridges simultaneously to develop an accurate model. The equilibrium differential equations are derived using the extended Hamilton’s principle and Galerkin decomposition is implemented to discretize the nonlinear equations. To improve the accuracy of the solution by consuming the lowest computations, the number of mode shapes and the size of voltage increments are optimized. Herein, free vibrations of the nano-beam incorporating influences of different system parameters are analyzed. Determining the critical voltage of thermomechanical piezoelectric nano-systems is the principal objective of this paper. This adjustable thermomechanical model is able to control the stability conditions at different temperatures. The results are expected to be instrumental in the analysis, design, and operation of several NEMS.

## Theoretical Model

**Figure 1:**
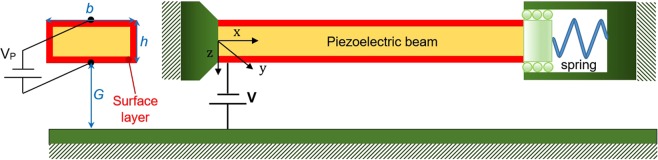
Schematic of a piezoelectric controllable nano-system with fixed-sliding boundary conditions under Casimir and electrostatic effects.

The schematic of a piezoelectric nano-beam fabricated from a movable rectangular arm and a stationary plate as the substrate is represented in Fig. 1. The deformable electrode deflects toward the substrate due to the attractions of Casimir and electric forces. Furthermore, the piezobeam is actuated by the direct current *V*_P_ as the polarization voltage, which is applied along the thickness of the nano-beam and leads to a load in the longitudinal direction^33^. Such a configuration as an adjustable system is useful in several nano-bridges, tunable filters, thermal gates, cooling devices, and variable switches^31,35,36^. In this case, the boundary conditions (BCs) of the structure are such that the beam does not tolerate any traction along its neutral axis. There is not any rotation or vertical displacement at the right end.

### Strain, potential, and kinetic energy

Based on the thermo-elasticity mechanics, the strain energy of a thermo-sensitive beam *U*_*t*_ is

1 $${U}_{t}=-\,\frac{EA\Delta T}{2}{\alpha }_{t}{\int }_{0}^{L}{(\frac{\partial w(x,t)}{\partial x})}^{2}dx,$$

where *A*, *E*, Δ*Τ*, and *α*_*t*_ are the rectangular cross-sectional area, bulk Young’s modulus of the beam or modulus of elasticity, temperature variation, and thermal expansion coefficient, respectively. Furthermore, *w* is the beam displacement along *z* coordinate axis.

It should be mentioned that all the symbols used in this work are defined in the Nomenclature. The nonlinear curvature effect should be considered when the deformable beam undergoes distortion due to the external force. Therefore, we obtain the strain as

2 $${\xi }_{xx}(x,t)=\frac{ds-dx}{dx},$$

where *s* and *ξ*_*xx*_ are the actual length arched beam during deflection and beam axial strain at its neutral axis, respectively.

It should be mentioned that the energy of the linear spring can be given as

3 $${U}_{k}=\frac{1}{2}{K}_{s}{u}^{2}(x,t),$$

where *U*_*k*_, *K*_*s*_, and *u* are the spring potential energy, beam displacement along *x* coordinate axis, and linked spring stiffness, respectively.

The nonlinear curvature *ζ* is stated as^37^

4 $$\zeta (x,t)=\frac{d\theta }{ds},$$

where *θ* is the angle of arched element.

By taking the nonlinear curvature of the piezobeam into account, both of axial strain *ε*_*xx*_ and stress *σ*_*xx*_ can be expressed as^19,38^

5 $${\varepsilon }_{xx}(x,t)={\xi }_{xx}(x,t)-z\zeta (x,t);\,{\rm{all}}\,{\rm{other}}\,{\varepsilon }_{ij}=0,$$

6 $${\sigma }_{xx}(x,t)=E{\varepsilon }_{xx}(x,t)-{e}_{31}{E}_{z};\,{\rm{all}}\,{\rm{other}}\,{\sigma }_{ij}=0,$$

where *e*_31_ is the transversal piezoelectric coefficient (−6.4 C.m^−2^).

To model the electrostatic response of the piezobeam, the energy of the piezobeam *U*_*p*_ is stated as^38^

7 $${U}_{P}=-\,{e}_{31}\frac{{V}_{P}}{h}\frac{A}{2}{\int }_{0}^{L}{(\frac{\partial w}{\partial x})}^{2}dx,$$

where *h*, *L*, and *V*_P_ are the beam thickness, beam length, and the piezovoltage between top and bottom surfaces, respectively.

Moreover, the moment of the nano-beam by considering bulk and surface layer is expressed as

8 $$M={\int }_{A}{\sigma }_{xx}zdz+{\int }_{S}({\tau }_{0}+{E}^{s}{\varepsilon }_{xx}^{s})zdz=-\,\zeta (x,t)(\frac{Eb{h}^{3}}{12}+\frac{{E}^{s}b{h}^{2}}{2}+\frac{{E}^{s}{h}^{3}}{6}),$$

where *b*, *E*^*s*^, *τ*_0_, and *ε*_*xx*_^*s*^ are the beam width, surface Young’s modulus around the beam, surface strain around the beam (beam surface layer strain), and residual stress of the beam surface area, respectively.

Therefore, the energy of the nano-beam is derived as

9 $${U}_{m}=\frac{{E}_{eff}{I}_{eff}}{2}{\int }_{0}^{L}{\zeta }^{2}(x,t)dx,$$

where *U*_*m*_ and *E*_*eff*_ *I*_*eff*_ are the mechanical strain energy and effective Young’s modulus and second moment of area of beam (bulk and surface layer), respectively.

The energy due to the surface layer tension *U*_*s*_ is obtained as

10 $${U}_{s}={\tau }_{0}b{\int }_{0}^{L}\zeta (x,t)w(x,t)\,dx$$

Due to the nonclassical modified couple stress theory (MCST), the energy of a beam in the submicron-scale is given by^17^

11 $${U}_{l,{\rm{MCS}}}=\frac{bhE{\ell }^{2}}{4(1+\nu )}{\int }_{0}^{L}{(\frac{{\partial }^{2}w(x,t)}{{\partial }^{2}x})}^{2}dx,$$

where *U*_*l*_, *ℓ*, and *ν* are the strain energy due to material size, material nano-scale factor or size-dependent parameter according to MCST, and Poisson’s ratio, respectively.

According to another nonclassical SGT^17^, the energy of a nano-beam is^39^

12 $${U}_{l,{\rm{SG}}}={\int }_{0}^{L}[\begin{array}{c}\frac{bhE}{4(1+\nu )}(2{l}_{0}^{2}+\frac{8{l}_{1}^{2}}{15}+{l}_{2}^{2}){(\frac{{\partial }^{2}w(x,t)}{\partial {x}^{2}})}^{2}\\ \,+\,\frac{b{h}^{3}E}{48(1+\nu )}(2{l}_{0}^{2}+0.8{l}_{1}^{2}){(\frac{{\partial }^{3}w(x,t)}{\partial {x}^{3}})}^{2}\end{array}]dx,$$

where *ℓ*_i_ (i = 0, 1, 2) are the additional length parameters (dilatation, deviatoric stretch, and rotation gradients).

Furthermore, the kinetic energy of the beam is

13 $${E}_{k}=\frac{1}{2}{\int }_{0}^{L}\rho A{(\frac{\partial w(x,t)}{\partial t})}^{2}dx,$$

where *ρ* is the beam density.

### External work

In general, the work carried out by possible attractions can be given by

14 $${W}_{ext}={\int }_{0}^{L}{F}_{ext}w(x,t)dx,$$

where *W*_*ext*_ and *F*_*ext*_ are the external work and distributed external force (per unit length), respectively.

Considering two parallel plates (moveable electrode and substrate) without considering FFC, the electric attraction can be estimated as^12,25^

15 $${F}_{els}=\frac{{\varepsilon }_{0}b{V}^{2}}{2{(G-w(x,t))}^{2}},$$

where *G*, *V*, and *ε*_0_ are the initial separation or gap, external applied DC voltage or electric potential difference, and electrical permittivity constant of free space vacuum (8.854 × 10^−12^ F.m^−1^), respectively.

Although, Parallel-Plates (PP) model for electric force is the most common configuration in numerous applications; NEMS designers are turning the focus to the electric FFC. In order to complement this effect, different corrections can be suggested to the pure electric expression. Taking into account the simplest as well as the most extensively used FFC in the literature, which is known as Palmer’s (PM) model, the electrostatic attraction can be given by^12^

16 $${F}_{els,PM}=\frac{{\varepsilon }_{0}b{V}^{2}}{2{(G-w(x,t))}^{2}}+\frac{0.65{\varepsilon }_{0}{V}^{2}}{2(G-w(x,t))}.$$

In addition, Mejis-Fokkema (MF) model is another famous one, which can adjust the electrostatic force as follows^13^:

17 $${F}_{els,MF}=\frac{{\varepsilon }_{0}b{V}^{2}}{2{(G-w(x,t))}^{2}}(1+0.265{(\frac{G-w(x,t)}{b})}^{0.75}+0.53\frac{h}{b}{(\frac{G-w(x,t)}{b})}^{0.5}),$$

where the effects of both beam width and thickness have been reflected.

On the other hand, Casimir regime is important in nano-devices, which can be disregarded beyond this scale. Casimir force is given by^40^

18 $${F}_{cas}=\frac{{\pi }^{2}bc{h}_{p}}{240{(G-w(x,t))}^{4}},$$

where *c* and *h*_*p*_ are the light speed (2.9979 × 10^8^ m.s^−1^) and reduced Planck’s constant (1.055 × 10^−34^ J.s), respectively.

The studied nano-manipulator is under both Casimir *F*_*cas*_ and electric *F*_*els*_ forces by considering FFC, so the performed work by the mentioned forces is given by

19 $${W}_{ext}={\int }_{0}^{L}({\int }_{0}^{w}(\{\begin{array}{c}{F}_{els}\\ {F}_{els,PP}\\ {F}_{els,MF}\end{array}+{F}_{cas})dw(x,t))dx.$$

### Governing equations according to MCST and SGT

To derive the nonlinear EOM of the presented nano-manipulator, the well-known extended Hamilton’s principle^41^ is applied as

20 $$\delta {\int }_{0}^{t}({U}_{t}+{U}_{k}+{U}_{p}+{U}_{m}+{U}_{s}+{U}_{l}-{E}_{k}-{W}_{ext})dt=0.$$

By replacing the potential, kinetic, and strain energy plus the performed work by other (corrected electrostatic as well as Casimir) forces considering the related supplementary relations into Eq. (20), using the variational approach, the following PDE for the piezoelectric size-dependent nano-beam can be obtained

21 $$\begin{array}{c}\{\begin{array}{c}\frac{bhE}{2(1+\nu )}{\ell }^{2}\frac{{{\rm{\partial }}}^{4}w}{{\rm{\partial }}{x}^{4}}(forMCST)\\ \frac{b{h}^{3}E}{24(1+\nu )}(2{l}_{0}^{2}+\frac{8{l}_{1}^{2}}{10})\frac{{d}^{6}w}{d{x}^{6}}+\frac{bhE}{2(1+\nu )}(2{l}_{0}^{2}+8{l}_{1}^{2}/15+{l}_{2}^{2})\frac{{{\rm{\partial }}}^{4}w}{{\rm{\partial }}{x}^{4}}(forSGT)\end{array}\\ +\,(E\frac{b{h}^{3}}{12}+{E}_{s}(\frac{b{h}^{2}}{2}+\frac{{h}^{3}}{6}))[\frac{{{\rm{\partial }}}^{4}w}{{\rm{\partial }}{x}^{4}}+\frac{{\rm{\partial }}}{{\rm{\partial }}x}(\frac{{\rm{\partial }}w}{{\rm{\partial }}x}\frac{{\rm{\partial }}}{{\rm{\partial }}x}(\frac{{{\rm{\partial }}}^{2}w}{{\rm{\partial }}{x}^{2}}\frac{{\rm{\partial }}w}{{\rm{\partial }}x}))]-b{\tau }_{0}\frac{{{\rm{\partial }}}^{2}w}{{\rm{\partial }}{x}^{2}}(4+{(\frac{{\rm{\partial }}w}{{\rm{\partial }}x})}^{2})\\ +\,(Ebh\varDelta T{\alpha }_{T}+{e}_{31}{V}_{{\rm{P}}}b-sign(\theta )\frac{{K}_{s}}{2}{\int }_{0}^{L}{(\frac{{\rm{\partial }}w}{{\rm{\partial }}x})}^{2}dx)\frac{{{\rm{\partial }}}^{2}w}{{\rm{\partial }}{x}^{2}}+\rho bh\frac{{{\rm{\partial }}}^{2}w}{{\rm{\partial }}{t}^{2}}={F}_{ext}.\end{array}$$

The boundary conditions (BC) of the considered system are

22 $$w(0)=w(L)=0,dw(0)/dx=dw(L)/dx=0.$$

The mentioned BC are not sufficient for the strain gradient model. Accordingly, they are written as

23 $$\begin{array}{c}w(0)=w(L)=0,dw(0)/dx=dw(L)/dx=0,\\ {d}^{2}w(0)/d{x}^{2}={d}^{2}w(L)/d{x}^{2}=0,OR\,{d}^{3}w(0)/d{x}^{3}={d}^{3}w(L)/d{x}^{3}=0.\end{array}$$

For the sake of the scalability of the problem, the nonlinear equations of motion are considered in the dimensionless form as

24 $$\begin{array}{c}\chi =\frac{x}{L},\,\varpi =\frac{w}{G},\,\tau =\frac{ht}{2{L}^{2}}\sqrt{\frac{E}{3\rho }},\,\phi =\frac{G}{b},\,\xi =\frac{{G}^{2}}{{L}^{2}},\,\beta =\frac{{L}^{2}}{{h}^{2}},\,\eta =\frac{2{E}^{s}}{E}(\frac{3}{h}+\frac{1}{b}),\\ \iota =\frac{6{\ell }^{2}}{(1+\nu ){h}^{2}},\,{\iota }_{0}=\frac{{l}_{0}^{2}}{(1+\nu ){L}^{2}},\,{\iota }_{1}=\frac{0.4{l}_{1}^{2}}{(1+\nu ){L}^{2}},\,{\iota }_{2}=\frac{6{l}_{2}^{2}}{(1+\nu ){h}^{2}},\,\lambda =\frac{48{\tau }_{0}{L}^{2}}{E{h}^{3}},\\ \vartheta =\frac{12{L}^{2}\varDelta T{\alpha }_{T}}{{h}^{2}},\,\varUpsilon =\frac{12{L}^{2}{e}_{31}{V}_{{\rm{P}}}}{E{h}^{3}},\,{c}_{cas}=\frac{{\pi }^{2}{h}_{p}c{L}^{4}}{20{h}^{3}{G}^{5}E},\\ \kappa =sign(\varDelta T)\frac{6L{G}^{2}{K}_{s}}{Eb{h}^{3}}{\int }_{0}^{L}{(\frac{\partial \varpi }{\partial \chi })}^{2}d\chi ,\,\upsilon =\frac{V{L}^{2}}{hG}\sqrt{\frac{6{\varepsilon }_{0}}{hGE}},\end{array}$$

*χ*: dimensionless length according to beam length (*x*/*L*); *ϖ*: dimensionless beam midpoint displacement (*w/G*); *τ*: dimensionless time; *ϕ*: ratio of initial gap to beam width (*G/b*); *ξ*: square ratio of initial gap to beam length; *β*: square ratio of beam length to thickness; *η*: surface elasticity dimensionless parameter; *ι*: material size dimensionless parameter according to MCST; *ι*_*i*_ (*i* = 0, 1, 2): length dimensionless parameters according to SGT; *λ*: residual surface-induces normal stress dimensionless parameter; *ϑ*: thermal expansion dimensionless parameter; ϒ: piezoelectric voltage dimensionless parameter; *c*_*cas*_: Casimir dimensionless coefficient; *κ*: dimensionless spring coefficient; *υ*: dimensionless voltage.

Substituting the dimensionless terms Eq. (24) into Eq. (21), the equations of motion are derived from the equilibrium equation as

25 $$\begin{array}{c}\{\begin{array}{c}\iota \frac{{\partial }^{4}\varpi }{\partial {\chi }^{4}}\\ ({\iota }_{0}+{\iota }_{1})\frac{{\partial }^{6}\varpi }{\partial {\chi }^{6}}+(12{\iota }_{0}\beta +8{\iota }_{1}\beta +{\iota }_{2})\frac{{\partial }^{4}\varpi }{\partial {\chi }^{4}}\end{array}+(1+\eta )\frac{{\partial }^{4}\varpi }{\partial {\chi }^{4}}\\ +\,(\vartheta +\varUpsilon -\kappa -4\lambda )\frac{{\partial }^{2}\varpi }{\partial {\chi }^{2}}+\xi ((1+\eta )[\frac{\partial }{\partial \chi }(\frac{\partial \varpi }{\partial \chi }\frac{\partial }{\partial \chi }(\frac{{\partial }^{2}\varpi }{\partial {\chi }^{2}}\frac{\partial \varpi }{\partial \chi }))]-\lambda {(\frac{\partial \varpi }{\partial \chi })}^{2}\frac{{\partial }^{2}\varpi }{\partial {\chi }^{2}})\\ +\,\frac{{\partial }^{2}\varpi }{\partial {\tau }^{2}}=\frac{{\upsilon }^{2}}{{(1-\varpi )}^{2}}\{\begin{array}{c}1\\ 1+0.65\phi (1-\varpi )\\ 1+0.265{(\phi (1-\varpi ))}^{0.75}+0.53\sqrt{\xi /\beta }{\phi }^{1.5}{(1-\varpi )}^{0.5}\end{array}+\frac{{c}_{cas}}{{(1-\varpi )}^{4}}.\end{array}$$

## Solution Methodology

In general, analytical techniques are not able to solve the nonlinear differential equations of excited thermal piezo NEMS. For that reason, the governing equation of motion is better to be linearized and solved by numerical methods. In this research, a computational method called SSLM is expended to estimate threshold characteristics. In the following, the term *ϖ* will be considered as a linear combination of a number of modes or a set of basic functions, which are defined independently from each other. In this condition, the approximate solution of the studied structure will be constructed as

26 $$\varpi ={A}^{{\rm{T}}}\varphi =\mathop{\sum }\limits_{i=1}^{N}{A}_{i}{\varsigma }_{i}(\chi ),$$

where *Α*_*i*_ is the amplitude factor of the beam transverse displacement and *φ* is the non-normalized system mode shapes vector includes a set of harmonic functions *ς*_*i*_, e.g. as following for MCST case^42^

27 $${\varsigma }_{i}={B}_{i}((\sin ({\delta }_{i}\chi )-\,\sinh ({\delta }_{i}\chi ))\frac{\cosh \,{\delta }_{i}-\,\cos \,{\delta }_{i}}{\sinh \,{\delta }_{i}-\,\sin \,{\delta }_{i}}+\,\cosh ({\delta }_{i}\chi )-\,\cos ({\delta }_{i}\chi )),$$

where terms *B*_*i*_ signifies the displacement scaling and the values *δ*_*i*_ are the characteristic equation roots. For SGT also different solutions have been presented in several papers^39,43^.

Having the Eq. (27) and replacing the Eq. (26) into (25), multiplying both sides by *φ*(*χ*), ultimately the governing equation is converted as

28 $$n=m\ddot{B}+\{\begin{array}{c}\iota {K}_{1}B\\ (({\iota }_{0}+{\iota }_{1}){K}_{0}+(12{\iota }_{0}\beta +8{\iota }_{1}\beta +{\iota }_{2}){K}_{1})B\end{array}+(\begin{array}{c}(1+\eta ){K}_{1}+(\vartheta +{\Upsilon }-\kappa -4\lambda ){K}_{3}\\ \,+\,\xi (2(1+\eta ){K}_{2}-\lambda {K}_{4})\end{array})B,$$

where beam stiffness expressions *K*_*i*_ (*i* = 0, 1, 2, 3, 4) and the beam inertia expression *m* are obtained as

29 $$\begin{array}{c}{K}_{0}={\int }_{0}^{1}\frac{{\partial }^{3}\varphi }{\partial {\chi }^{3}}\frac{{\partial }^{3}{\varphi }^{T}}{\partial {\chi }^{3}}d\chi ,\,{K}_{1}={\int }_{0}^{1}\frac{{\partial }^{2}\varphi }{\partial {\chi }^{2}}\frac{{\partial }^{2}{\varphi }^{T}}{\partial {\chi }^{2}}d\chi ,\,{K}_{2}={\int }_{0}^{1}\frac{\partial \varphi }{\partial \chi }\frac{{\partial }^{2}{\varphi }^{T}}{\partial {\chi }^{2}}B\frac{\partial {\varphi }^{T}}{\partial \chi }B\frac{{\partial }^{2}{\varphi }^{T}}{\partial {\chi }^{2}}d\chi ,\\ {K}_{3}={\int }_{0}^{1}\varphi \frac{{\partial }^{2}{\varphi }^{T}}{\partial {\chi }^{2}}d\chi ,\,{K}_{4}={\int }_{0}^{1}\varphi \frac{\partial {\varphi }^{T}}{\partial \chi }B{B}^{T}\frac{\partial \varphi }{\partial \chi }\frac{{\partial }^{2}\varphi }{\partial {\chi }^{2}}d\chi ,\,m={\int }_{0}^{1}\varphi {\varphi }^{T}d\chi ,\end{array}$$

and

30 $$n={\int }_{0}^{1}(\frac{{\upsilon }^{2}}{{(1-B\varphi )}^{2}}\{\begin{array}{c}1\\ 1+0.65\phi (1-B\varphi )\\ 1+0.265{(\phi (1-B\varphi ))}^{0.75}+0.53\sqrt{\xi /\beta }{\phi }^{1.5}{(1-B\varphi )}^{0.5}\end{array}+\frac{{c}_{cas}}{{(1-B\varphi )}^{4}})\varphi d\chi .$$

Having the Eq. (28), the total stiffness of the nano-beam is expressed as

31 $$\begin{array}{c}K=\{\begin{array}{c}\iota {K}_{1}\\ ({\iota }_{0}+{\iota }_{1}){K}_{0}+(12{\iota }_{0}\beta +8{\iota }_{1}\beta +{\iota }_{2}){K}_{1}\end{array}+(1+\eta ){K}_{1}\\ \,+\,6\xi (1+\eta ){K}_{2}+(\vartheta +\varUpsilon -\kappa -4\lambda ){K}_{3}-3\xi \lambda {K}_{4}-dn(B)/dB,\end{array}$$

where

32 $$\frac{dn(B)}{dB}={\int }_{0}^{1}(\frac{2{\upsilon }^{2}}{{(1-B\varphi )}^{3}}\{\begin{array}{c}1\\ 1+0.325\phi (1-B\varphi )\\ 1+\frac{0.1656{\phi }^{0.75}}{{(1-B\varphi )}^{-0.75}}+\frac{0.3975\sqrt{\xi /\beta }{\phi }^{1.5}}{{(1-B\varphi )}^{-0.5}}\end{array}+\frac{4{c}_{cas}}{{(1-B\varphi )}^{5}})\,{\varphi }^{2}d\chi .$$

### The static analysis

In actuated electromechanical systems, the movable arm cannot tolerate any external voltage. In this case, the response of nano-devices is achievable by considering Eq. (33) as

33 $$(\{\begin{array}{c}\iota {K}_{1}\\ ({\iota }_{0}+{\iota }_{1}){K}_{0}+(12{\iota }_{0}\beta +8{\iota }_{1}\beta +{\iota }_{2}){K}_{1}\end{array}+(\begin{array}{c}(1+\eta ){K}_{1}+(\vartheta +\varUpsilon -\kappa -4\lambda ){K}_{3}\\ \,+\,\xi (2(1+\eta ){K}_{2}-\lambda {K}_{4})\end{array}))B=n.$$

At the unstable conditions, the system stiffness will become singular or det(*K*) equals zero when the instability happens^44^. This method prepares a useful and practical way of computing the instability condition of the structures.

The nonlinear motion equations are solved numerically. In this research work, SSLM is employed^45^, and instability characteristics will be determined. Because of the nonlinear treatment of considered nano-beam, linearizing initial states may be a source of errors. To minimize the possible errors, SSLM is considered to increase the external electrostatic actuation slightly. Due to sufficient small increments of the external actuation, the beam deflection will also increase slightly. As a result, we will be able to model the nonlinear behavior of the thermal nano-switch accurately. Moreover, by accounting Taylor’s expansions, accuracy necessities are obtained. Considering Eq. (33), the equation of equilibrium at the *i*th step is derived as

34 $$(\{\begin{array}{c}\iota {K}_{1}^{i}\\ ({\iota }_{0}+{\iota }_{1}){K}_{0}^{i}+(12{\iota }_{0}\beta +8{\iota }_{1}\beta +{\iota }_{2}){K}_{1}^{i}\end{array}+(\begin{array}{c}(1+\eta ){K}_{1}^{i}+(\vartheta +\varUpsilon -\kappa -4\lambda ){K}_{3}^{i}\\ \,+\,\xi (2(1+\eta ){K}_{2}^{i}-\lambda {K}_{4}^{i})\end{array}))B={n}^{i}.$$

As an example, the term *n*^*i*^ is achieved from Eq. (30) as

35 $${n}^{i}={\int }_{0}^{1}(\frac{{({\upsilon }^{i})}^{2}}{{(1-B{\varphi }^{i})}^{2}}\{\begin{array}{c}1\\ 1+0.65\phi (1-B{\varphi }^{i})\\ 1+\frac{0.265{\phi }^{0.75}}{{(1-B{\varphi }^{i})}^{-0.75}}+\frac{0.53\sqrt{\xi /\beta }{\phi }^{1.5}}{{(1-B{\varphi }^{i})}^{-0.5}}\end{array}+\frac{{c}_{cas}}{{(1-B{\varphi }^{i})}^{4}}){\varphi }^{i}d\chi .$$

### The eigenvalue problem

To obtain the system frequencies at different voltages, the static solution can be substituted into the characteristic equation for the corresponding eigenvalues. To this end, the frequency of the structure equals zero, when the pull-in takes place. Consequently, the frequency of the manipulator can be achieved via the following equation.

36 $$\det \,(K-m{\omega }^{2})=0,$$

where “det” refers to the determinant operator and the terms *K* and *m* are presented in Eqs. (31) and (29), respectively. Accordingly, by considering the dynamic governing equation (Eq. (28)) as well as Eq. (36), the *i*-th step will be derived as

37 $${m}^{i}\ddot{B}+\{\begin{array}{c}\iota {K}_{1}^{i}\\ ({\iota }_{0}+{\iota }_{1}){K}_{0}^{i}+(12{\iota }_{0}\beta +8{\iota }_{1}\beta +{\iota }_{2}){K}_{1}^{i}\end{array}B+(\begin{array}{c}(1+\eta ){K}_{1}^{i}+(\vartheta +\varUpsilon -\kappa -4\lambda ){K}_{3}^{i}\\ +\xi (2(1+\eta ){K}_{2}^{i}-\lambda {K}_{4}^{i})\end{array})B={n}^{i}.$$

## Results and Discussion

The taken step length of the solution procedure can meaningfully influence the accuracy of obtaining responses. Firstly, we will try to determine an appropriate length to increase the external electrostatic actuation in SSLM. For this purpose, responses of the prepared system in ref. ^35^ considering different voltage lengths are listed in Table 1. It can be concluded that the obtained responses will be converged by taking an adequately small step length into account.

**Table 1:** Convergence of system responses considering different voltage increases.

Voltage increase steps (V)	0.2	0.02	0.002	0.0002
Instability voltage (V)	12.4	11.88	11.791	11.7140
Error ratios (%)	5.98	1.45	0.66	—

In order to make validation, we have compared the threshold voltages with the experimental^46^, analytical ^26^, and numerical^27^ results reported in the literature for beams with different length (Table 2). The width and thickness of the deformable beam are 50 and 1 μm, respectively, and the initial gap is 3 μm. Furthermore, the modulus of elasticity and Poison’s ratio are 169 GPa and 0.6, respectively. As it can be seen, the results obtained from our model show a good correlation with experiments validating its high accuracy.

**Table 2:** Comparison of the pull-in voltage with experimental, analytical and numerical data.

	Voltage (L = 250 μm)	Voltage (L = 350 μm)
Experiment^46^	39.5 V	20.20 V
Analytic^26^	39.4 V	20.10 V
Numeric^27^	39.3 V	20.07 V
Present study	39.6 V	20.40 V

As another validation, a fixed-sliding micro-beam in the attendance of electrostatic is modeled and pull-in characteristics are obtained and compared with a micro-structure with similar BCs (Table 3). Koochi *et al*.^35^ examined a fixed-sliding micro-beam using the Modified Adomain decomposition and numerical solution as well as a lumped model, which assumes the uniform external forces along the beam. The available theoretical results, which are provided in Table 4, illustrate that the responses of the performed analysis are in an appropriate agreement with available data in the open literature.

**Table 3:** Comparison the obtained results with the available results^35^.

	Modified Adomian decomposition	Numerical solution	Lumped model	SSLM (Present study)
Critical voltage parameter	7.11	6.99	6.32	7.014
Critical deflection parameter	0.45	0.43	0.36	0.439

**Table 4:** Properties and dimensions of the proposed manipulator.

	E	v	α_T_	b	H	L	G
Value	68.5 GPa	0.33	−9.5 × 10^−6^ K^−1^	0.2 μm	0.1 μm	12 μm	60 nm

The dimensions and constants employed to model the nano-system are mentioned in Table 4. More details and explanations will be discussed in the subsequent section.

### Static analysis

The relationships between the mid-beam deflections versus the voltage will be displayed in this section. Based on the figures, mid-beam deflections increase with an increase in the value of the applied voltage. In general, the actuated nano-beam is not able to endure relatively considerable voltages. Note that all reported results presented in this section (Static analysis) are according to MCST unless specified otherwise.

**Figure 2:**
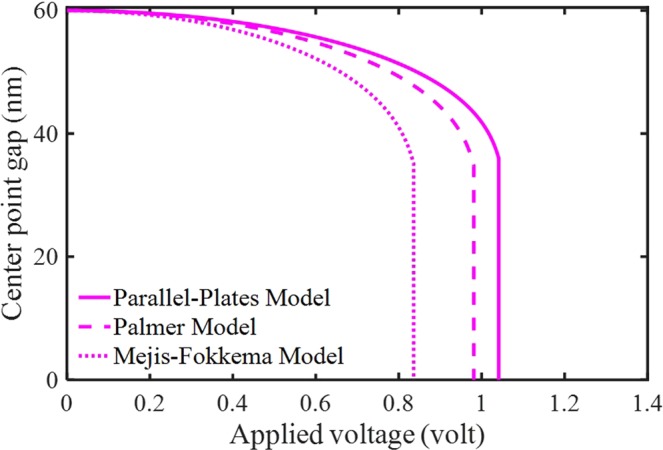
The impacts of the electrostatic force and different fringing-fields on the system behavior.

Figure 2 shows the static defection variation with respect to different expressions of the fringing correction when the DC voltage increases from zero. Two well-known models for FFC, which are PM and MF models in addition to the electrostatic PP model for the electric actuation, are assumed to correct the distributed electric force. It is recognized that the impacts of the FFC on the system stability are remarkable. The figures display that considering the FFC makes the electrode softer and causes the decline of instability voltages. Furthermore, it can be seen that the effects of FFC are more significant by taking MF than PM model. The obtained results completely agree with the results of clamped-clamped nano-beams for different types of electric FFC reported by Ouakad^20^. He found that the responses of MF model are generally in better agreement with the finite element method (FEM) and experimental data.

**Figure 3:**
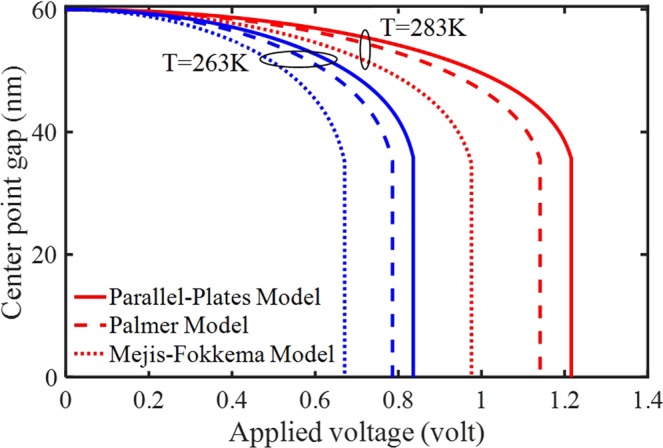
The impacts of the thermal force and different fringing-fields on the system behavior.

Figure 3 displays the deflection of the electrode, including the thermal variations and FFC (*K*_S_ = 1 N/m). In this case, we consider the actuated system with (*F*_*els,PP*_ and *F*_*els,MF*_) and without (*F*_*els*_) fringing effects at the temperatures of 263 K and 283 K. It can be concluded that the effects of FFC on the stability are remarkable at different temperatures or when the system operates in thermally fluctuating conditions. The effect of the linear spring on the structural behavior should be explained with respect to the sign of the thermal expansion coefficient (Table 4). It is also seen that the nano-system becomes more stable (i.e., higher pull-in voltage is obtained) with increasing the temperature. This is due to the negative coefficient of the thermal expansion (i.e., the beam starts shrinking while heated) that causes increasing the tensile strength by applying a load in the *x*-direction. Moreover, the curves illustrate that an increase in the ambient temperature makes the system harder and increases the contributions of electric force corrections. It means that the effect of FFC becomes greater at higher temperatures and differences between instability voltages will increase.

**Figure 4:**
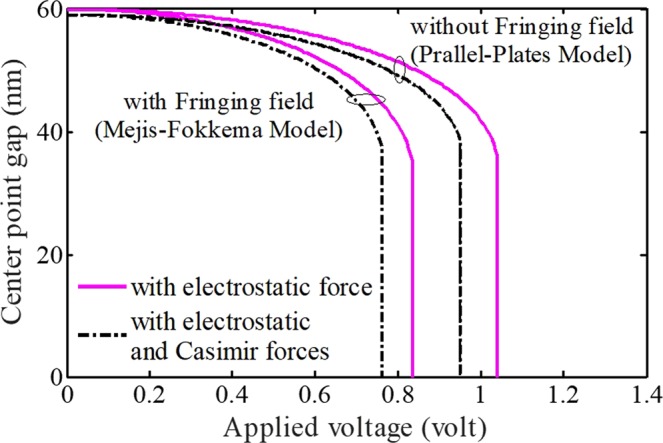
The impacts of Casimir force and Mejis-Fokkema fringing-field on the system.

In Fig. 4, the impact of Casimir force with (considering MF model) and without taking FFC (assuming PP model) on the system behavior are presented. Generally, the instability condition or pull-in phenomenon happens with a delay in nano-systems without considering Casimir force. In addition, when there is no electric force, the deformable electrode endures a primary displacement due to Casimir effect. On the other hand, the influence of this force on the critical applied voltage becomes more obvious in the Parallel-Plates model. Accordingly, differences between the instability voltages decrease slightly with considering electric FFC. Moreover, by using the relation ${c}_{cas}={\pi }^{2}{h}_{p}c{L}^{4}/20{h}^{3}{G}^{5}E$, it can be understood that the impact of Casimir force enhances with an increase in the nano-beam length, unlike the thickness. This also specifies that the impact of Casimir on the system behavior is more considerable for slender electrodes.

Comparing the obtained graphs confirms that FFC is vital to be considered in evaluating the behavior of nano-switches. It can be computed in a relative error of almost 24–25% in predicting the instability parameters of the MEMS when neglecting this effect. Therefore, we have found reasonable agreement between the responses of this dimensionless nano-system and the numerically calculated values for arch MEMS in Ref. ^20^.

**Figure 5:**
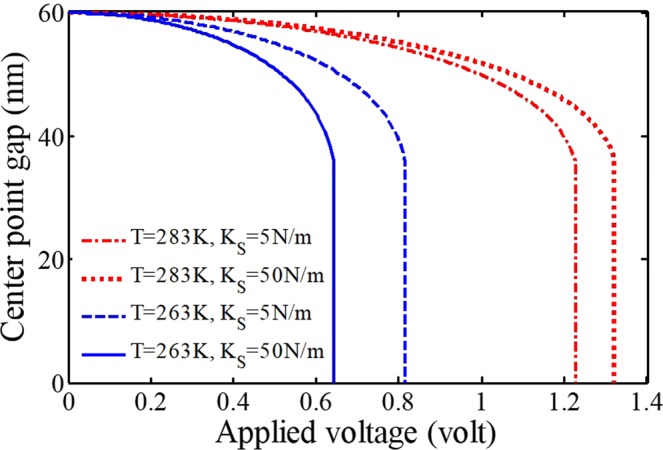
The impacts of the thermal force and spring stiffness on the system behavior.

Figure 5 illustrates the variations of the mid-beam at different amounts of spring stiffness vs. the external voltage. Here, as the spring stiffness tends to zero, there exists no thermal force in this configuration. Therefore, the thermal effect is meaningless without considering the spring in such beams with the fixed-sliding BCs (since a fixed-sliding beam like a cantilever one is capable of moving in the longitudinal axis, the temperature variation results in changing its length and it will be free of stress). On the other hand, as the spring stiffness tends to ∞, the responses become close to those for a clamped-clamped block. The influence of spring stiffness is more considerable at lower temperatures due to the difference between the pull-in voltages of blue curves, which should be explained regarding the thermal expansion coefficient. Finally, as a practical point, it is notable that the influence of temperature variations increases while the stiffness enhances, so the difference between the pull-in voltages becomes greater in devices with doubly clamped BCs.

**Figure 6:**
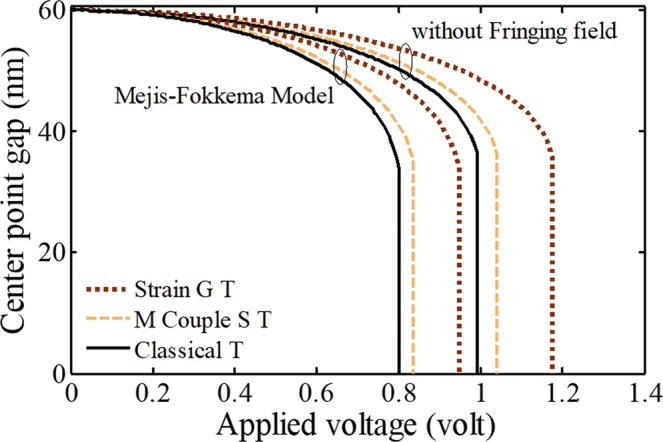
The impacts of different size theories and fringing-fields on the system behavior.

Figure 6 shows the relationship between the curves and the excitation including/excluding the effects of FFC using different size theories. To investigate the impact of higher-order material size parameters, the achieved results have been compared using MCST and SGT. First, note that in this research, the higher-order bending rigidity of material is assumed 21 nm. As a result, the modified couple stress length-scale parameter is 15 nm^17,47^. Assuming three equal strain gradient length-scale parameters^39^, these parameters will be equal to 9 nm^43,48^. The results demonstrate that both MCST and SGT make the beam to be harder and cause an increase in the threshold voltage.

**Figure 7:**
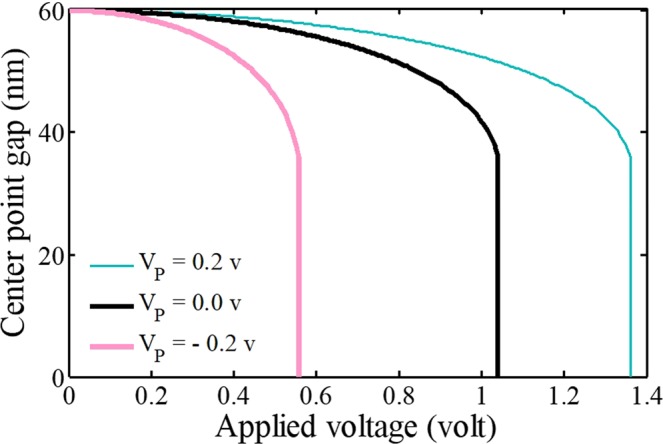
The impact of the piezoelectric voltage on the system behavior.

In Fig. 7, it can be found that the piezovoltage affects the system response significantly by changing the structural behavior. Based on the application, the piezovoltage can compress or stretch the beam by considering the polarity. The results show that the positive actuation increases the instability voltage by increasing the beam stiffness where the negative polarity produces a compression force in the *x*-direction. It is comprehended that piezoexcitations shift the equilibrium manifold and consequently the instability voltage. The results agree well with the reported ones in clamped-clamped piezobased bridges^32,33^.

**Figure 8:**
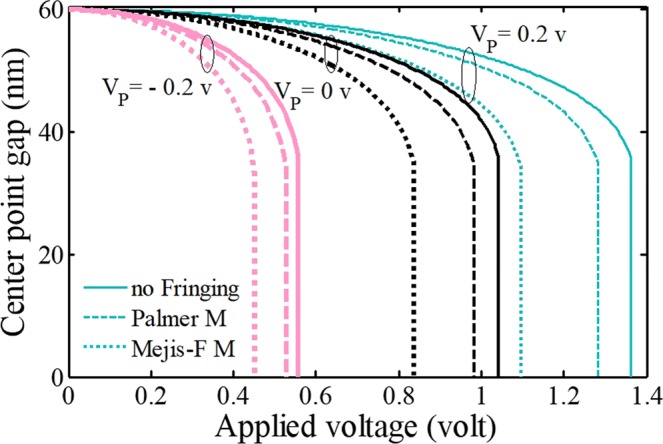
The impacts of the piezoelectric voltage and fringing-fields on the system behavior.

The relationships between the mid-beam vs. the electrostatic excitation by considering different FFC models with both positive and negative piezoexcitation are shown in Fig. 8. It can be observed that the impact of piezoelectricity changes by taking different models to consider FFC. Generally, as the piezoelectric voltage increases, the difference between the results of FFC models becomes more considerable. Consequently, differences between the instability voltages in FFC models are increased by considering the positive piezoelectric excitation. On the other hand, differences between the results of piezovoltage will increase as the negative polarity considered.

It is interesting to note that by tuning the value of piezoexcitation, the bridge behavior is changeable, so the pull-in voltage is controllable. As a result, we can increase the stability range or decrease the critical voltage by taking positive or negative piezoelectric actuation. Therefore, improving the performance of several nano-switches are possible by considering piezomaterials and applying the piezovoltage as an important design parameter in numerous applications.

### Dynamic analysis

Here, the vibrations of the rectangular electromechanical nano-system will be analyzed. It should be noted that for more accurate investigations of each parameter, these assumptions are taken into account unless specified otherwise (T = 273 K, *λ* = 0, *η* = 0, *ι* = 0, *ι*_*i*_ = 0, *V*_P_ = 0, *ϕ* = 0, and *c*_cas_ = 0). In these cases, the system frequency decreases with an increase in the external voltage at first. After that, the system response tends to 0 promptly, when the DC voltage goes to a maximum amount (near the critical value).

**Figure 9:**
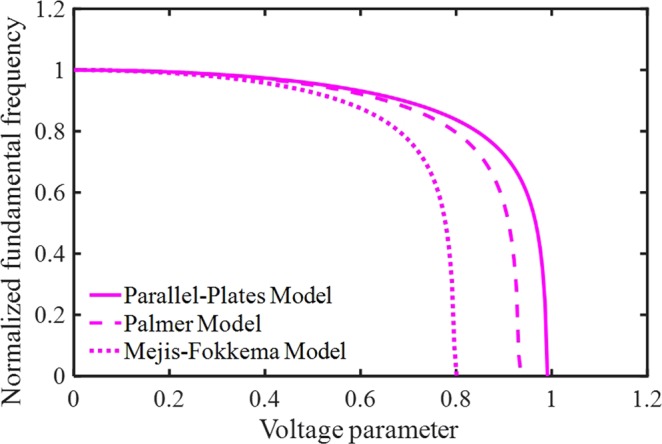
The impacts of different fringing-fields on the system dynamic behavior.

Figure 9 shows the relationships between the mid-beam and the voltage parameter by considering all PP, PM, and MF models. It can be concluded, taking FFC affects pull-in characteristics more than system frequencies. In addition, the impacts of the electrostatic force corrections on the nano-system frequency become more obvious with increasing the voltage gradually. The responses for system frequencies are qualitatively agreeable by accounting numerical results of Ouakad^20^, including the electric fringing-fields effect (all models). Finally, note that without applying the DC voltage, there is no difference between the response frequencies with/without accounting FFC (Fig. 9). It means that the start points of curves are the same in this figure unlike other figures of dynamic behavior.

**Figure 10:**
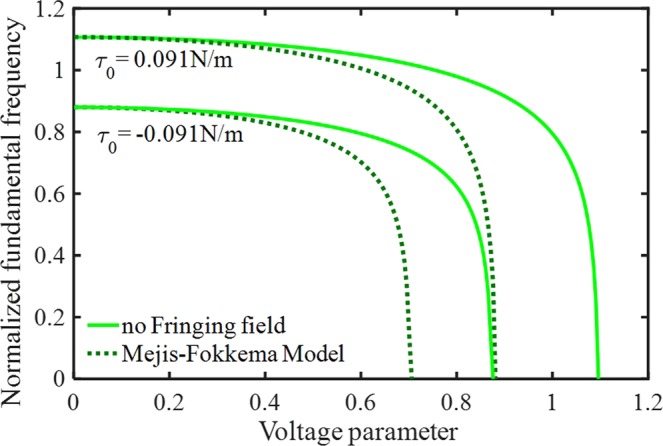
The impacts of residual surface stress on the system dynamic behavior.

The impact of the residual tension in the surface layer on the nano-system frequency is shown in Fig. 10. It is comprehended that system frequencies and instability voltages change with variations of the tension. Here, response frequencies and critical voltages increase by considering positive surface stress. On the other hand, the values for the residual surface stress nano-materials may be negative. In this case, the results are exactly the opposite of the positive one, so considering the negative stress makes the fixed-sliding beams soften. Moreover, using the relation $\lambda =24{\tau }_{0}{L}^{2}/E{h}^{3}$, it is recognized the influence of residual surface tension reduces with decreasing the nano-beam length and increasing the thickness. Therefore, this molecular surface effect will be more dominant in the slender nano-structures. It is necessary to mention that the effect of FFC becomes more significant by considering positive term; however, the difference between the positive and negative stress decreases, including FFC.

**Figure 11:**
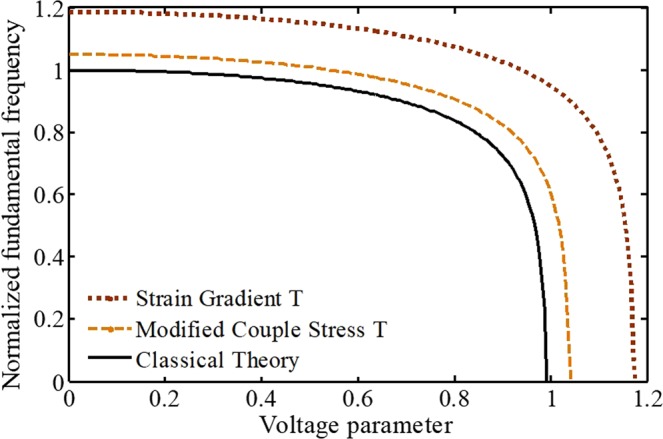
The impacts of different nonclassical size theories on the system dynamic behavior.

Figure 11 displays the effect of different nonclassical size theories (MCST and SGT) on the dynamic behavior and response frequencies of the nano-beam. The curves illustrate that by considering the length-scale parameters, we have a stiffer nano-system. Therefore, both system frequencies and dynamic voltage will be increased by taking nonclassical theories into account. Moreover, differences between the results of SGT and classical theory is more considerable. Ultimately, from relations of material size parameters, it is found that the influence of size parameters becomes weak by increasing the beam thickness.

The obtained results of this research allow a better understanding of the instability of the nano-beam based tiny devices and can guide scientists to improve their general performance, appropriately. Moreover, the outcomes of this study explain the causes of differences obtained in the literature when comparing the responses of the nano-system without considering modified models including nonclassical theories, small-scale effects, and external force corrections to experimental data. There is still plenty of exploration to be discovered regarding the reliability of electro-thermo-mechanical nano-systems. Forthcoming investigations need to be established, including modelling the impacts of out‐of‐plane electrodes arrangement on the behavior of nano-devices with different BCs by considering both size and surface effects, which should be verified experimentally.

## Conclusion

In order to investigate the pull-in phenomenon of adjustable intelligent systems, a nano-beam with a sliding end connected to spring was taken into account and a thermomechanical model was presented. In the model, the impacts of the surface layer, molecular forces, size-dependency, piezoelectricity, and temperature variations were included with consideration of the nonlinear curvature. Two famous models for the electric fringing corrections, which are PM and MF models, were assumed to correct the distributed electrostatic force. The PDEs were derived by the extended Hamilton’s principle. Galerkin decomposition was implemented to discretize the nonlinear equations. To improve the accuracy of responses by spending affordable computational calculations, the number of mode shapes and the size of voltage increments were optimized. Afterward, the structural behavior of the nano-system under Casimir and corrected electric attractions were analyzed numerically. Finally, the system vibrations with regard to the environment temperature, surface layer, and material size theories (MCST and SGT) were studied.

The obtained results reveal that the classical theory predicts lower values for instability voltages and system frequencies. It was also shown that considering the electric FFC is essential for accurate modelling of the nano-switches behavior. Moreover, the effect of MF model is more remarkable than the other one. In addition, this effect causes a significant change at higher temperatures as well as in cases of positive residual surface stress and positive piezoelectric voltage. As a practical point, the effect of temperature variation increases while the stiffness of the connected spring enhances, so differences between the critical voltage at different temperatures becomes greater in clamped-clamped beams. Finally, it should be mentioned that the piezoexcitation could be employed as a design parameter to improve the controllability and increase the performance range of adjustable smart devices.

## References

[1] Araneo R, Falconi C (2013). Lateral bending of tapered piezo-semiconductive nanostructures for ultra-sensitive mechanical force to voltage conversion. Nanotechnology.

[2] Jang J (2015). A microelectromechanical system artificial basilar membrane based on a piezoelectric cantilever array and its characterization using an animal model. Scientific Reports.

[3] Ramini AH, Hajjaj AZ, Younis MI (2016). Tunable resonators for nonlinear modal interactions. Scientific Reports.

[4] SoltanRezaee, M., Ghazavi, M.-R. & Najafi, A. in 853: Modelling, Simulation and Identification / 854: Intelligent Systems and Control.July 19-20 edn (eds PZJ Chen & MH Hamza) 73-79 (Acta Press).

[5] Zolfagharian A, Darzi M, Ghasemi S (2017). Analysis of nano droplet dynamics with various sphericities using efficient computational techniques. Journal of Central South University.

[6] Valipour P, Zaersabet H, Hatami M, Zolfagharian A, Ghasemi S (2017). Numerical study on polymer nanofibers with electrically charged jet of viscoelastic fluid in electrospinning process. Journal of Central South University.

[7] Hosseini R (2017). Parameter identification of partially covered piezoelectric cantilever power scavenger based on the coupled distributed parameter solution. International Journal of Smart and Nano Materials.

[8] SoltanRezaee M, Ghazavi M-R, Najafi A (2018). Parametric resonances for torsional vibration of excited rotating machineries with nonconstant velocity joints. Journal of Vibration and Control.

[9] Nasri-Nasrabadi B (2018). An electroactive polymer composite with reinforced bending strength, based on tubular micro carbonized-cellulose. Chemical Engineering Journal.

[10] Falconi C (2019). Piezoelectric nanotransducers. Nano Energy.

[11] SoltanRezaee M, Bodaghi M, Farrokhabadi A (2019). A thermosensitive electromechanical model for detecting biological particles. Scientific Reports.

[12] Gupta, R. K. Electrostatic pull-in test structure design for mechanical property characterization of microelectromechanical systems (MEMS) *Ph.D. thesis*, (1997).

[13] Batra R, Porfiri M, Spinello D (2006). Capacitance estimate for electrostatically actuated narrow microbeams. Micro & Nano Letters.

[14] Guo J-G, Zhao Y-P (2004). Influence of van der Waals and Casimir forces on electrostatic torsional actuators. Journal of Microelectromechanical Systems.

[15] Klimchitskaya G, Mohideen U, Mostepanenko V (2000). Casimir and van der Waals forces between two plates or a sphere (lens) above a plate made of real metals. Physical Review A.

[16] Yang F, Chong A, Lam DC, Tong P (2002). Couple stress based strain gradient theory for elasticity. International Journal of Solids and Structures.

[17] Lam DC, Yang F, Chong A, Wang J, Tong P (2003). Experiments and theory in strain gradient elasticity. Journal of the Mechanics and Physics of Solids.

[18] Gurtin ME, Murdoch AI (1975). A continuum theory of elastic material surfaces. Archive for Rational Mechanics and Analysis.

[19] He J, Lilley CM (2008). Surface effect on the elastic behavior of static bending nanowires. Nano Letters.

[20] Ouakad HM (2018). Electrostatic fringing-fields effects on the structural behavior of MEMS shallow arches. Microsystem Technologies.

[21] Esfahani S, Khadem SE, Mamaghani AE (2019). Size-dependent nonlinear vibration of an electrostatic nanobeam actuator considering surface effects and inter-molecular interactions. International Journal of Mechanics and Materials in Design.

[22] Guo J-G, Zhao Y-P (2005). The size-dependent elastic properties of nanofilms with surface effects. Journal of Applied Physics.

[23] SoltanRezaee M, Bodaghi M, Farrokhabadi A, Hedayati R (2019). Nonlinear stability analysis of piecewise actuated piezoelectric microstructures. International Journal of Mechanical Sciences.

[24] Ramezani A, Alasty A, Akbari J (2007). Closed-form solutions of the pull-in instability in nano-cantilevers under electrostatic and intermolecular surface forces. International Journal of Solids and Structures.

[25] Batra RC, Porfiri M, Spinello D (2006). Electromechanical model of electrically actuated narrow microbeams. Journal of Microelectromechanical systems.

[26] Rokni H, Lu W (2013). Surface and thermal effects on the pull-in behavior of doubly-clamped graphene nanoribbons under electrostatic and Casimir loads. Journal of Applied Mechanics.

[27] Tavakolian F, Farrokhabadi A, Mirzaei M (2017). Pull-in instability of double clamped microbeams under dispersion forces in the presence of thermal and residual stress effects using nonlocal elasticity theory. Microsystem Technologies.

[28] SoltanRezaee M, Ghazavi MR (2017). Thermal, size and surface effects on the nonlinear pull-in of small-scale piezoelectric actuators. Smart Materials and Structures.

[29] Pradiptya I, Ouakad HM (2018). Thermal effect on the dynamic behavior of nanobeam resonator assuming size-dependent higher-order strain gradient theory. Microsystem Technologies.

[30] SoltanRezaee M, Afrashi M, Rahmanian S (2018). Vibration analysis of thermoelastic nano-wires under Coulomb and dispersion forces. International Journal of Mechanical Sciences.

[31] Tavakolian F, Farrokhabadi A, SoltanRezaee M, Rahmanian S (2019). Dynamic pull-in of thermal cantilever nanoswitches subjected to dispersion and axial forces using nonlocal elasticity theory. Microsystem Technologies.

[32] Pourkiaee SM, Khadem SE, Shahgholi M (2016). Parametric resonances of an electrically actuated piezoelectric nanobeam resonator considering surface effects and intermolecular interactions. Nonlinear Dynamics.

[33] Pourkiaee SM, Khadem SE, Shahgholi M (2017). Nonlinear vibration and stability analysis of an electrically actuated piezoelectric nanobeam considering surface effects and intermolecular interactions. Journal of Vibration and Control.

[34] Nikpourian A, Ghazavi MR, Azizi S (2018). On the nonlinear dynamics of a piezoelectrically tuned micro-resonator based on non-classical elasticity theories. International Journal of Mechanics and Materials in Design.

[35] Koochi A, Kazemi AS, Beni YT, Yekrangi A, Abadyan M (2010). Theoretical study of the effect of Casimir attraction on the pull-in behavior of beam-type NEMS using modified Adomian method. Physica E: Low-dimensional Systems and Nanostructures.

[36] Beni YT, Koochi A, Abadyan M (2011). Theoretical study of the effect of Casimir force, elastic boundary conditions and size dependency on the pull-in instability of beam-type NEMS. Physica E: Low-dimensional Systems and Nanostructures.

[37] Hodges DH (1984). Proper definition of curvature in nonlinear beam kinematics. AIAA Journal.

[38] Gheshlaghi B, Hasheminejad SM (2012). Vibration analysis of piezoelectric nanowires with surface and small scale effects. Current Applied Physics.

[39] Kong S, Zhou S, Nie Z, Wang K (2009). Static and dynamic analysis of micro beams based on strain gradient elasticity theory. International Journal of Engineering Science.

[40] Israelachvili, J. N. (Academic Press, London, 1992).

[41] He J-H (2017). Hamilton’s principle for dynamical elasticity. Applied Mathematics Letters.

[42] Hodges, D. H. & Pierce, G. A. Introduction to structural dynamics and aeroelasticity. **Vol. 15** (cambridge university press, 2011).

[43] Kahrobaiyan M, Asghari M, Ahmadian M (2013). Strain gradient beam element. Finite Elements in Analysis and Design.

[44] Wang K, Wang B (2015). A general model for nano-cantilever switches with consideration of surface effects and nonlinear curvature. Physica E: Low-dimensional Systems and Nanostructures.

[45] Rashvand K, Rezazadeh G, Mobki H, Ghayesh MH (2013). On the size-dependent behavior of a capacitive circular micro-plate considering the variable length-scale parameter. International Journal of Mechanical Sciences.

[46] Osterberg PM, Senturia SD (1997). M-Test: a test chip for MEMS material property measurement using electrostatically actuated test structures. Journal of Microelectromechanical Systems.

[47] Park S, Gao X (2006). Bernoulli–Euler beam model based on a modified couple stress theory. Journal of Micromechanics and Microengineering.

[48] Dehrouyeh-Semnani AM (2015). A comment on “Static and dynamic analysis of micro beams based on strain gradient elasticity theory”[Int. J. Eng. Sci. 47 (2009) 487–498]. International Journal of Engineering Science.

